# Volatility of Secondary Organic Aerosol from β-Caryophyllene Ozonolysis over a Wide Tropospheric Temperature Range

**DOI:** 10.1021/acs.est.3c01151

**Published:** 2023-06-07

**Authors:** Linyu Gao, Angela Buchholz, Zijun Li, Junwei Song, Magdalena Vallon, Feng Jiang, Ottmar Möhler, Thomas Leisner, Harald Saathoff

**Affiliations:** † Institute of Meteorology and Climate Research, Karlsruhe Institute of Technology, Karlsruhe 76344, Germany; ‡ Institute of Geography and Geoecology, Working Group for Environmental Mineralogy and Environmental System Analysis, Karlsruhe Institute of Technology, Karlsruhe 76131, Germany; § Department of Technical Physics, University of Eastern Finland, Kuopio 70210, Finland; ∥ Institute of Environmental Physics, Heidelberg University, Heidelberg 69120, Germany; ⊥ International Laboratory for Air Quality and Health, School of Earth and Atmospheric Sciences, Queensland University of Technology, Brisbane, QLD 4001, Australia

**Keywords:** volatility, secondary organic aerosol (SOA), positive matrix factorization (PMF), β-caryophyllene, temperature dependence

## Abstract

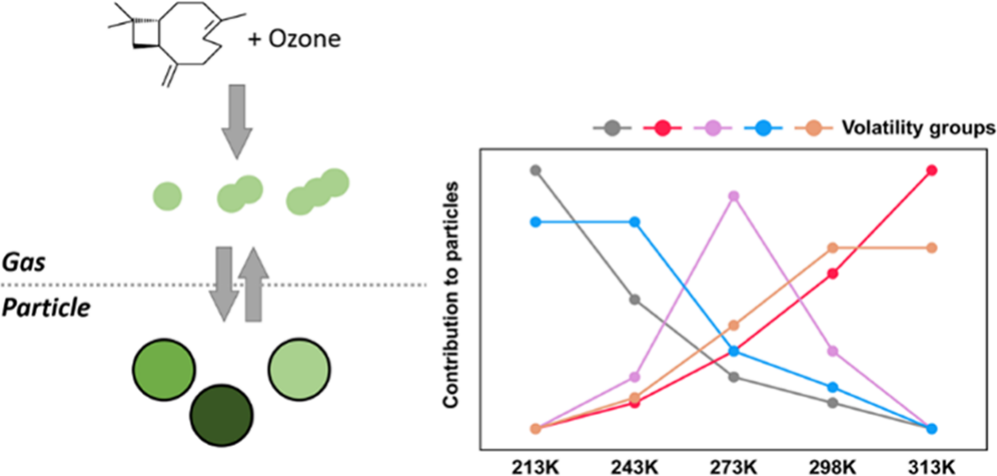

We investigated secondary organic aerosol (SOA) from β-caryophyllene oxidation generated over a wide tropospheric temperature range (213–313 K) from ozonolysis. Positive matrix factorization (PMF) was used to deconvolute the desorption data (thermograms) of SOA products detected by a chemical ionization mass spectrometer (FIGAERO-CIMS). A nonmonotonic dependence of particle volatility (saturation concentration at 298 K, *C*_298K_^*^) on formation temperature (213–313 K) was observed, primarily due to temperature-dependent formation pathways of β-caryophyllene oxidation products. The PMF analysis grouped detected ions into 11 compound groups (factors) with characteristic volatility. These compound groups act as indicators for the underlying SOA formation mechanisms. Their different temperature responses revealed that the relevant chemical pathways (e.g., autoxidation, oligomer formation, and isomer formation) had distinct optimal temperatures between 213 and 313 K, significantly beyond the effect of temperature-dependent partitioning. Furthermore, PMF-resolved volatility groups were compared with volatility basis set (VBS) distributions based on different vapor pressure estimation methods. The variation of the volatilities predicted by different methods is affected by highly oxygenated molecules, isomers, and thermal decomposition of oligomers with long carbon chains. This work distinguishes multiple isomers and identifies compound groups of varying volatilities, providing new insights into the temperature-dependent formation mechanisms of β-caryophyllene-derived SOA particles.

## Abstract 2

This work investigates the temperature-dependent volatility of β-caryophyllene-derived SOA particles, providing new insights into the formation mechanism of new SOA particles.

## Introduction

Secondary organic aerosol (SOA) has adverse effects on air quality, human health, and the climate.^1−4^ Oxidation products of biogenic volatile organic compounds (BVOCs) are the main contributors to SOA mass by gas-to-particle partitioning after their oxidation in the gas phase.^5−7^ Volatility is a key physicochemical property determining the condensation and evaporation of, e.g., organic compounds. Thus, volatility determines the atmospheric fate of organic molecules and is important for understanding SOA formation and growth.^8^

However, it still remains unclear how environmental temperature impacts the formation of biogenic SOA particles in the atmosphere. Reduced temperatures lower the saturation vapor pressures (*V*_p_) of compounds according to the Clausius–Clapeyron relation, which would drive organic compounds into the particle phase.^8^ On the other hand, it was found that for the ozonolysis of α-pinene^9,10^ and β-caryophyllene,^11^ higher temperatures strongly affect gas-phase unimolecular reaction rates and increase the generation of more oxidized compounds with lower *V*_p_.^6^ Thus, the temperature can have opposite impacts on the condensation of less oxidized compounds and the formation of highly oxygenated organic molecules (HOMs). The interplay between these two aspects increases the complexity of SOA formation and complicates the predictions of particle volatility in different seasons and/or altitudes.^12^

Currently, the mostly used method to derive the volatility of organic compounds from mass spectrometry data is the parameterization of the detected elemental formulas.^13−15^ Parameterization methods omit the existence of isomers, i.e., one volatility value is assigned for each elemental formula, while volatilities of isomers may vary by orders of magnitude owing to their different functionalities and molecular structures.^16,17^ For the chemical ionization time-of-flight mass spectrometer (TOFMS) with filter inlet for gases and AEROsols (FIGAERO-CIMS) measurements, the thermal desorption behavior (thermograms) of the detected ions correlates with their *V*_p_. If an elemental formula represents only one compound, the thermogram would be monomodal and the *T*_max_ value directly relates to its *V*_p_.^18,19^ If multiple isomers are present and/or other compounds thermally decompose to this sum formula, multiple modes may be observed and a single *T*_max_ is no longer representative for the overall volatility.^18,20^ A newly extended approach based on positive matrix factorization (PMF) analysis^13,21^ helps us to distinguish isomers and thermal decomposition compounds in mass spectrometry data by deconvoluting the thermal desorption profiles of ions.

Recently, sesquiterpenes (C_15_H_24_) have gained more attention due to their importance in contributing to the overall SOA mass yield from, e.g., Scots pine emissions and the potential underestimation of their contribution to the global SOA mass.^22−29^ A recent modeling study showed that the global SOA burden can be enhanced by 48% relative to the base case when including sesquiterpenes into the model.^30^ β-Caryophyllene has the largest emissions among all bicyclic sesquiterpenes, which retain their large carbon chains throughout the oxidation process, and thus it acts as the proxy of sesquiterpenes in modeling studies on the estimation of global biogenic SOA mass.^31,32^ Due to its high reactivity toward ozone^33,34^ and low volatilities of its oxidation products, such as its atmospheric tracer β-caryophyllinic acid (C_14_H_22_O_4_),^35^ β-caryophyllene plays an important role in SOA formation and particle growth in local cases.^22,25,36,37^ Strong temperature dependence was found in the chemical composition of SOA from β-caryophyllene oxidation with abundant dimers/oligomers (e.g., C_28–30_H_44–48_O_5–9_ and C_41–44_H_62–66_O_9–11_) at 213–243 K and higher oxidized monomers (e.g., C_14–15_H_22–24_O_3–7_) at 298–313 K.^11^ However, to date, there is still a lack of understanding on the interplay between the ozonolysis chemistry and the phase partitioning at different temperatures during the formation of β-caryophyllene SOA. Determining volatilities of many β-caryophyllene oxidation products formed at different temperatures is still challenging.

In this work, we applied PMF to revisit a previously published data set of β-caryophyllene ozonolysis SOA formed between 213 and 313 K^11^ to derive volatility information and provided indicators of temperature-dependent formation mechanisms as an example for other biogenic SOA. The use of PMF enables us to investigate the varying contributions of dimers/oligomers even if they thermally decompose during the measurements.

## Experimental Section

### Simulation Chamber Experiments

Ozonolysis experiments were performed in the 84.5 m^3^ aluminum Aerosol Interaction and Dynamics in the Atmosphere (AIDA) simulation chamber at the Karlsruhe Institute of Technology (KIT).^38,39^ The chamber operation, experimental conditions, and instrument setup for the campaign are described in the Supporting Information. Briefly, five β-caryophyllene oxidation experiments were separately conducted in the dark at 213, 243, 273, 298, and 313 K (see Table S1). The β-caryophyllene (98%, Carl Roth GmbH) concentration injected into the chamber was 1.6 ppbv at 243 K and 8–12 ppbv at 273–313 K. At 213 K, due to strong wall loss effects, β-caryophyllene was lost to the chamber wall before the ozone addition, preventing SOA formation. Thus, to generate particles in quantities comparable to other experiments, more β-caryophyllene was added subsequently after the ozone addition for the experiment at 213 K. Ozone (99.9999%) was typically in excess with concentrations of 290–320 ppb except for the 273 K experiment, where the initial ozone concentration was 73 ppbv. No hydroxyl radical scavenger was used in any experiments. Note that the β-caryophyllene concentration as well as its ratio to ozone varied between individual experiments. However, in all experiments, ozone was in a substantial excess compared to the β-caryophyllene concentration. Thus, the temperature is the dominant influence, e.g., for the formation of oligomers, instead of the varying precursor concentrations.

### Particle Measurement

An iodide adduct chemical ionization mass spectrometer (I^–^-CIMS) coupled with a Filter Inlet for Gas and AEROsol (FIGAERO)^18^ (Aerodyne Research Inc. & Tofwerk AG) was used to analyze the composition and volatility of SOA particles.

The particle data presented in this work stem from offline analysis. Particles were deposited on a Teflon filter (poly(tetrafluorethylene), PTFE, 1 μm, SKC Inc.) with a collection flow rate of 6.4 L min^–1^ for typically 5–10 min. Afterward, the collected samples were stored in a freezer (at −30 °C) and then were analyzed by the FIGAERO-CIMS using pure nitrogen (99.9999%, Basi Schöberl GmbH) as a carrier gas. The desorption temperature was ramped linearly from 25 to 200 °C for 15 min and then held near 200 °C for 20 min to ensure the evaporation of most compounds deposited on the filter. Data were collected at 1 Hz and averaged over 10 s for postprocessing. Raw data were analyzed using Tofware v3.1.2. Although the sensitivities of the iodide CIMS toward oxygenated compounds span about three orders of magnitude, we assumed uniform sensitivity for all compounds detected by FIGAERO-CIMS and only used signal intensity for the comparisons presented here. Note that we did not calculate concentrations except for β-caryophyllinic acid for which we were able to determine the sensitivity to be (2.4_–0.63_^+0.96^) cps/ppt, while the maximum sensitivity of the iodide CIMS has been determined at 22 cps/ppt.^40^ The limitations of the FIGAERO offline analysis are discussed in the SI.

Before each experiment, the chamber air was sampled and analyzed in the same fashion as the experimental samples to provide the background stemming from the chamber, filter matrix, and instrument. The mass spectra of background filter samples were subtracted from those of particle samples for the same experiments.

Additionally, the bulk chemical composition was monitored with a high-resolution time-of-flight aerosol mass spectrometer (HR-AMS, Aerodyne Research Inc.) in real time. High-resolution analysis of the elemental composition including the oxygen-to-carbon (O/C) and hydrogen-to-carbon (H/C) ratios^41^ was done with PIKA 1.20 C.^42,43^

### Deconvolution of Thermograms by Positive Matrix Factorization

The statistical method of PMF^21,44−46^ has recently been extended to study the volatilities of SOA constituents by analyzing the thermograms of individual molecules from FIGAERO-CIMS.^13−15^ Isomeric compounds and products from thermal decomposition of larger compounds that appear with the same molecular composition are grouped by their desorption behavior into PMF factors, which can then be compared between experiments. In this study, we used the constant error (CNerror)^13^ approach, which yielded the most interpretable results. The noise was calculated using the thermal desorption data at the end of the thermogram scans, and the CNerror was multiplied by 4 to improve the range of *Q*/*Q*_exp_^13^ values. The PMF analysis was calculated for 1–12 factors with three *f*_peak_ rotations^13^ from −0.5 to +0.5 using the FIGAERO Thermogram PMF Evaluation Tool (FiT PET v1.09 and PET v3.06, Dr. Angela Buchholz, private communication). All five β-caryophyllene SOA experiments were analyzed together and interpreted by the same set of factors. The diagnostics of the selected solution, error scheme, and comparison of different solutions are described in detail in Figures S6 and S7. A background factor (BG1) was identified, covering the remaining instrument background, which was not captured by the chamber blank measurements. The thermograms of C_15_H_24_O_3_ and C_15_H_24_O_4_ were excluded from the PMF analysis and resolved separately, as they have very high signal intensities but were not captured well by the PMF analysis.

### Three Volatility Estimation Methods

The volatility of individual compound is here expressed by the saturation concentration at a reference temperature of 298 K (*C*_298K_^*^, μg m^–3^). The *C*_298K_^*^ of a compound is related to its *V*_p_ as^47,48^
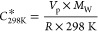
1where *M*_W_ is the molecular weight of a compound, g mol^–1^; *R* refers to the universal gas constant, 8.314 J K^–1^ mol^–1^. The saturation concentration of species at other temperatures (*C*_T_^*^) can be derived from *C*_298K_^*^ according to the Clausius–Clapeyron relation
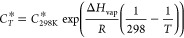
2where *T* is the experimental temperature in K; Δ*H*_vap_ is the evaporation enthalpy in kJ mol^–1^, which can be estimated by^49^
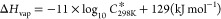
3For comparisons, we use the following volatility classes:^50,51^ ultralow VOC (ULVOC, log_10_ C* < −8.5, gray), extremely low VOC (ELVOC, −8.5 < log_10_* C*_298K_^*^ < −4.5, blue), low VOC (LVOC, −4.5 < log_10_* C*_298K_^*^ < −0.5, orange), semi-VOC (SVOC, −0.5 < log_10_* C*_298K_^*^ < −2.5, pink), intermediate VOC (IVOC, 2.5 < log_10_* C*_298K_^*^ < 6.5, green), and VOC (log_10_* C*_298K_^*^ > 6.5, yellow). The boundaries of the volatility classes are defined at 298 K and are shifted to the corresponding values at the formation temperatures using the Clausius–Clapeyron relation.^12^

Note that, to avoid any confusion in the description, the word “compound” hereafter refers to one unique constitute, and the word “ion” represents all of the detected isomers behind a common sum formula.

In this work, we used three approaches to determine *C*_298K_^*^: (1) the measured elemental formulas applying a parameterization using molecular corridors^8,52^ (“formula” method); (2) the correlation between effective vapor pressures and peak desorption temperatures of molecules^18,19,48,53^ (“*T*_max_ method”); (3) the correlation between effective vapor pressures and *T*_max_ of volatility groups after distinguishing isomers and thermal decomposition compounds identified by PMF (“PMF method”). The detailed description for each method and the calibration of *V*_p_ – *T*_max_ are given in the SI.

## Results and Discussion

### Bulk Particle Composition and Volatility

The average oxidation state of carbon (OS_C_) is a metric for the degree of oxidation of organic species.^54^ OS_C_ increases with the overall degree of oxidation. Since only C, H, and O atoms were relevant in this work, the OS_C_ was approximated as OS_C_ = 2 × O/C–H/C,^54^ with O/C and H/C being the ratios of oxygen and hydrogen to carbon. This simplified approach omits the existence of organic peroxides and may cause a small bias in the range of 0.1. The numbers of atoms for individual ions and the particle bulk were assigned from the measurements from FIGAERO-CIMS and HR-AMS, respectively. The OS_C_ of each individual ion was weighted with its signal fraction of the total particulate organic species to obtain the average OS_C_ for a FIGAERO-CIMS sample.

To have an overview of the SOA bulk composition at five different temperatures, the average O/C, H/C, and OS_C_ for each SOA particle sample are summarized in Table S3 (SI). The average O/C from AMS measurement increased from 0.21 to 0.45 with increasing temperatures from 213 to 313 K, indicating the higher oxidation degree of SOA particles with higher temperatures. HR-AMS measurements of particles formed at 213 to 273 K appear in the region between the slope of −2 (aldehydes/ketones) and −1 (carboxylic acids) in the van–Krevelen diagram (Figure S2). This suggests that the dominant functional groups in these SOA particles were ketone/aldehyde and carboxylic acids. The OS_C_ of these bulk particles was less than −1. The particles formed at 298 and 313 K had OS_C_ values higher than −1 and fell between the van–Krevelen slopes of −1 and 0, indicating a higher oxygen content and the presence of alcohol/peroxy groups. This trend is supported by the molecular chemical composition of the particle phase determined by FIGAERO-CIMS (Figure S3), where the monomers (carbon atoms ≤15, C_≤15_) were detected with higher oxygen content at 298–313 K than at 213–273 K, and dimers (C_16–30_) were only abundant at 213–243 K, as shown in Gao et al.^11^

**Figure 1:**
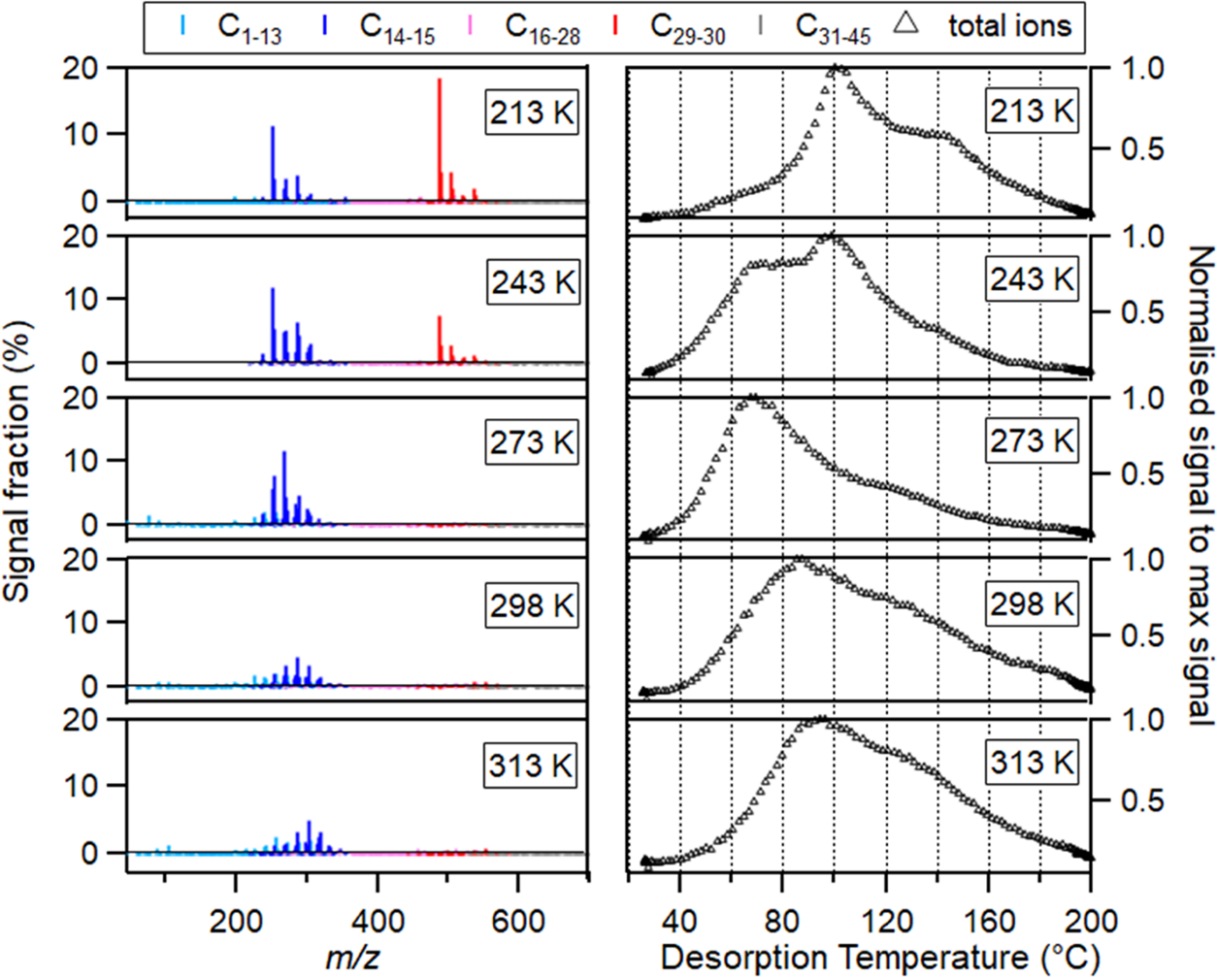
Mass spectra (left) and sum thermogram (right) of SOA (top to bottom) at 213, 243, 273, 298, and 313 K. In the left panel, compounds with different carbon numbers were colored as indicated in the legend. Monomers with 14–15 carbons and dimers with 29–30 carbon atoms are shown with positive values, and other molecules with 1–13 and 16–28 carbon atoms are represented by negative values to enhance readability. Mass spectra are reprinted with permission under the terms of the Creative Commons Attribution 4.0 License.^11^ Copyright 2022 Gao.

Figure 1 presents the mass spectra and the thermograms summed over the thermograms of all detected ions (thereafter “sum thermogram”). The sum thermograms differed in their shapes, and *T*_max_ values (thereafter “*T*_max,sum_”) for all SOA particles formed at varying temperatures. The *T*_max,sum_ first decreased from 97 °C (SOA_313K_) to 70 °C (SOA_273K_) and then increased to 101 °C (SOA_213K_) when the formation temperature was reduced from 313 to 213 K, also shown in Table S3 in the SI. The mass spectra show varying dominant products formed at different temperatures, indicating that the temperature dependence of chemical composition on the effective volatility of β-caryophyllene-derived SOA particles is nonmonotonic, leading to a counterintuitive behavior of *T*_max,sum_ of the sum thermograms. The multimodal thermograms of SOA_213K_ and SOA_243K_ and the broad thermogram shapes of SOA_273K_, SOA_298K_, and SOA_313K_ emphasize the complexity of the β-caryophyllene SOA composition as a function of formation temperatures. This complexity of thermograms indicated that the *T*_max,sum_ value of each sum thermogram was not necessarily representative of the overall particle volatility.

### Volatility and Chemistry of a Key Monomer Ion

**Figure 2:**
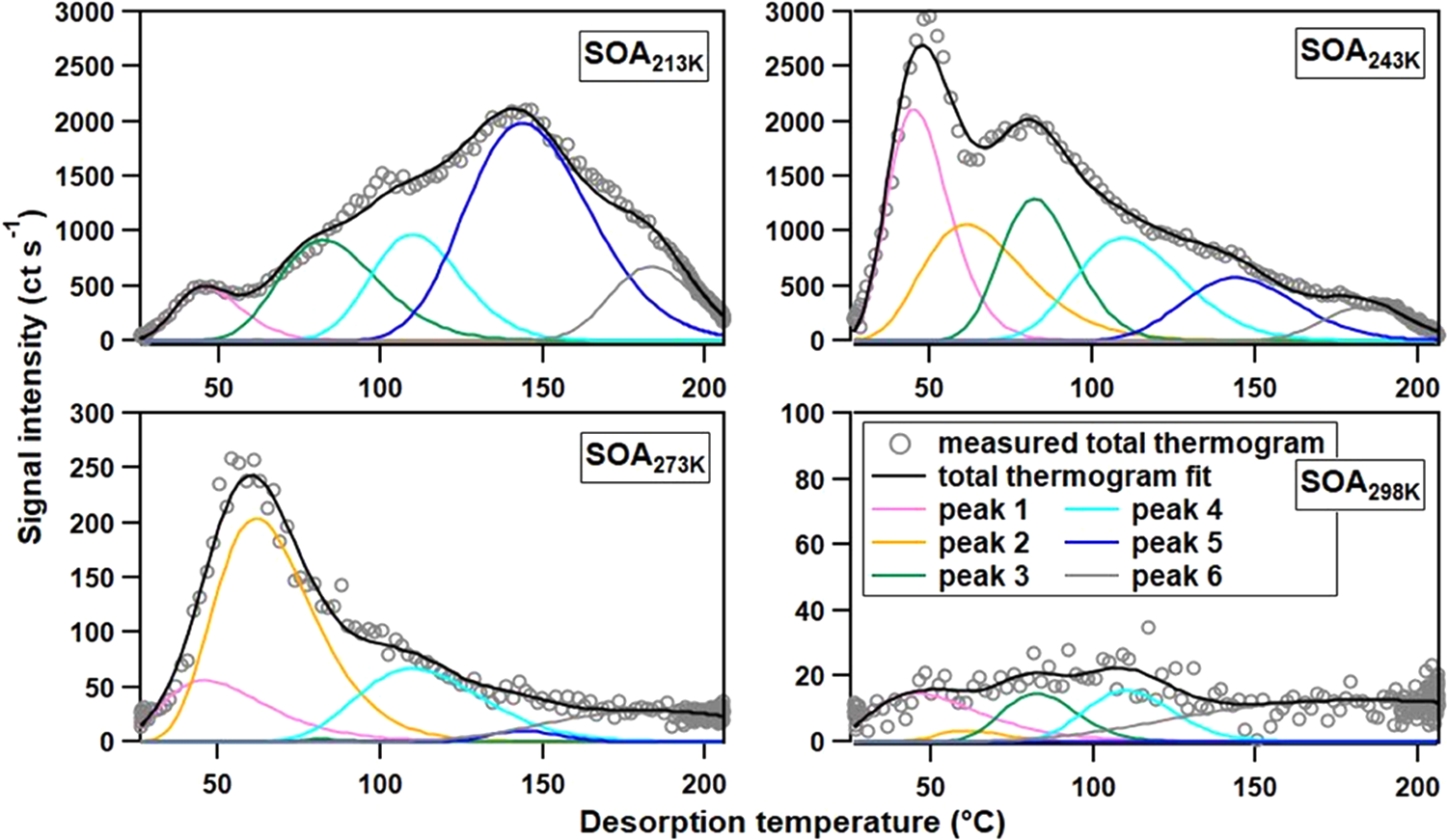
Fixed-peak Gaussian fit for C_15_H_24_O_3_I^–^ at a temperature (from top to bottom) of 213, 243, 273, and 298 K. Gray circles depict the measured data from FIGAERO-CIMS, while the black solid lines are the fitted total thermogram. Other colored solid lines show the individual compounds (fitted Gaussian peaks).

C_15_H_24_O_3_ is the most abundant ion for formation temperatures below 243 K. The ion thermograms are depicted in Figure 2. Two trends are visible: (1) the contribution of C_15_H_24_O_3_ in both particle and gas phases decreases with increasing formation temperatures from 243 to 313 K (Figure S4) and (2) the shape of the thermograms changes significantly. The particle phase concentration at 313 K was too low to yield a meaningful thermogram shape and is thus omitted from Figure 2.

For formation temperatures higher than 273 K, the major C_15_H_24_O_3_ compounds were desorbed below 100 °C during the thermal desorption. This indicates that the partitioning into the particle phase could be affected by the increasing formation temperature. However, the overall concentration of C_15_H_24_O_3_ in the gas phase also decreases at formation temperatures of 273 K or above (Figure S4), which suggests that the lower concentration of particulate C_15_H_24_O_3_ results mainly from the decreasing formation of C_15_H_24_O_3_. It is also possible that C_15_H_24_O_3_ is still formed at higher temperature but is then consumed by consecutive processes, e.g., condensed-phase reactions. The differences in the thermogram shapes for the SOA_213K_ cannot be explained by the changes in the overall concentration of C_15_H_24_O_3_ but must be linked to changes in the isomeric composition and/or the ratio between monomers and oligomers.

A careful inspection of the ion thermograms shows that for each experiment at least two peaks are clearly visible in the ion thermogram and that the *T*_max_ of these two peaks varies between experiments. By comparing the thermograms of all experiments, we conclude that up to six different compounds (isomers and/or decomposition products) contribute to the signal with this molecular mass or elemental composition. Hence, a set of six Gaussian peaks with manually chosen peak positions was used to fit the thermograms of C_15_H_24_O_3_I^–^ in all SOA samples (Figure 2), with a relative error between fitting and measured thermograms less than 2% for 243–298 K cases. Not all thermograms are composed of six modes, e.g., for SOA_213K_, compound 2 (*T*_max_ = 61 °C) did not contribute to the fitted thermogram. This indicates that the real compound represented by compound 2 may not exist in SOA_213K_. We acknowledge that the *T*_max_ values chosen for these 6 compounds will impact the fitting result. However, comparing multiple solutions with different *T*_max_ values showed that the overall interpretation presented below was not affected.

For C_15_H_24_O_3_, compounds fitted with *T*_max_ below ∼100 °C are assumed to be monomers since 100 °C is roughly the threshold temperature at which thermal decomposition may start to be relevant for carboxylic acid systems based on their estimated enthalpy of sublimation.^55^ In this range, three compounds contribute significantly to the total thermogram (compound 1 (*T*_max_ = 45 °C), compound 2 (*T*_max_ = 61 °C), and compound 3 (*T*_max_ = 82 °C) in SOA_213K_ and SOA_243K_). The observed change in the ratio between these three compounds cannot be explained by a shift toward lower volatility compounds with increasing formation temperature. Therefore, the formation pathway of the dominating isomer behind C_15_H_24_O_3_ changes at varying SOA formation temperatures.

In previous studies, two isomeric compounds (β-hydroxycaryophyllon aldehyde and β-caryophyllonic acid) were identified for C_15_H_24_O_3_ as early-stage oxidation products from β-caryophyllene ozonolysis.^34,35,56,57^ While it is not clear if these two compounds were indeed detected in our study, we can use these known molecular structures as examples of the types of compounds that may be produced. From the molecular structures, the expected *V*_p_ can be calculated with a group contribution method.^58,59^*V*_p_ (298 K) of 1.4 × 10^–4^ Pa (log *C*_sat_ (298 K) of −4.8) for the aldehyde and 3.8 × 10^–5^ Pa (log *C*_sat_ (298 K) of −5.4) for the acid were estimated. Their *V*_p_ is one order of magnitude different, which shows that isomeric compounds can have distinctly different volatilities and thus have different *T*_max_ values during the thermal desorption.

For the higher desorption temperatures, compound 5 (*T*_max_ = 144 °C) is dominating, especially in SOA_213K_, while it contributes less to the SOA formed at 243–298 K. Considering its *T*_max_, which is higher than expected for a compound with that sum formula, compound 5 is most likely a decomposition product of thermally unstable compounds with larger molecular weight, e.g., dimers or other oligomers. This suggests that the formation of dimers/oligomers, which can thermally fragment to C_15_H_24_O_3_, is favored at lower SOA formation temperatures. Thermal decomposition has been found to be a significant contributor to the total ion signal in monoterpene SOA,^48,60^ and here, we suggest that it is also important in β-caryophyllene SOA. Using the calibrated correlation between *V*_p_ and *T*_max_, we estimate the log_10_* C*_298K_^*^ for these six compounds as 2.7, 2.1, 1.2, 0.1, −1.3, and −2.8 μg m^–3^ in order of compound number. Thus, the C_15_H_24_O_3_ isomers span the LVOC and ULVOC ranges at 213–243 K and the SVOC and LVOC ranges at 273 K, while the potential decomposed oligomers are between the ULVOC and LVOC ranges at all SOA formation temperatures, revealing a high condensing potential for β-caryophyllene oxidation products.

The presence of multiple isomers was clear for C_15_H_24_O_3_ due to the distinct shape of the ion thermograms. For many other ions, it is also likely that isomers and thermal decomposition products are present, but the thermogram shapes were more difficult to interpret. Thus, manually choosing the true number of peaks and their *T*_max_ values became too subjective. Together with the larger number of ions in the data set, this made it infeasible to conduct a manual multipeak fit for every ion. Instead, we conducted a PMF analysis, which identifies correlations between the ion signals and can thus identify isomers or decomposition products with different volatilities within a single ion.

### PMF Factors as Indicators of the SOA Formation Mechanism

A 12-factor PMF solution was chosen as the optimal solution to explain the desorption behavior of the data set with the particle samples of the five formation temperatures (Figure S5). The factor composition differs between the experiments and could be divided into three groups: cold-temperature factors, intermediate-temperature factors, and warm-temperature factors, occurring at 213–243, 243–298, and 298–313 K, respectively. The two SOA samples in the cold cases are resolved by a similar factor pattern (C1–C6) dominated by monomers (C1, C2), dimers (C3, C4, C5), and thermal decomposition compounds from oligomers (C6) based on their thermal desorption behavior and factor chemical composition, indicating similar SOA formation processes at 213–243 K. The two SOA samples in the warm case are resolved by a totally different factor pattern (W1–W3) classified in the same way as mainly monomers (W1, W2) and dimers and/or oligomers with some thermal decomposition products (W3). Note that the properties of cold-temperature factors completely differ from those of the warm-temperature factors. For example, C1 has an average composition of C_14.2_H_24.0_ O_4.5_ and a *T*_max_ of 60 °C, while W1 has an average composition of C_13.8_ H_21.4_ O_5.8_ and a *T*_max_ of 85 °C, but both C1 and W1 are monomer factors. The properties of all PMF factors are described in Table 1, and the thermograms and modified Kroll diagram as well as mass spectra related to each PMF factor are shown in Figure S5. The detailed comparison of cold and warm patterns is described in the SI. The difference in factor composition between the warm and cold cases indicates the diversity of the chemical pathways and condensing processes involved in the SOA formation process in the different temperature regimes.

**Table 1:** Summary on the Average Molecular Formula, Molecular Weight (MW), O/C, OS_C_, and *T*_max_ of 12 PMF Factors

	factor number	molecular formula	*M*_W_ (g mol^–1^)	O/C	OS_C_	*T*_max_ (°C)
cold	C1	C_14.2_H_24.0_ O_4.5_	266	0.35	–0.99	60
	C2	C_14.9_ H_25.0_ O_5.6_	293	0.41	–0.85	85
	C3	C_24.7_ H_39.4_ O_6.1_	433	0.30	–1.00	105
	C4	C_27.3_ H_43.6_ O_5.4_	458	0.22	–1.16	95
	C5	C_24.8_ H_39.4_ O_7.0_	449	0.34	–0.93	120
	C6	C_28.4_ H_44.9_ O_6.5_	490	0.26	–1.08	145
warm	W1	C_13.8_ H_21.4_ O_5.8_	280	0.45	–0.66	85
	W2	C_15.0_ H_22.6_ O_6.8_	311	0.49	–0.53	100
	W3	C_22.9_ H_34.0_ O_7.8_	434	0.38	–0.74	135
intermediate	I1	C_13.5_ H_22.1_ O_5.1_	266	0.43	–0.80	70
	I2	C_19.8_ H_31.0_ O_6.1_	366	0.34	–0.9	125
background	BG1	C_14.3_ H_21.7_ O_5.3_	278	0.46	–0.61	N/A

**Figure 3:**
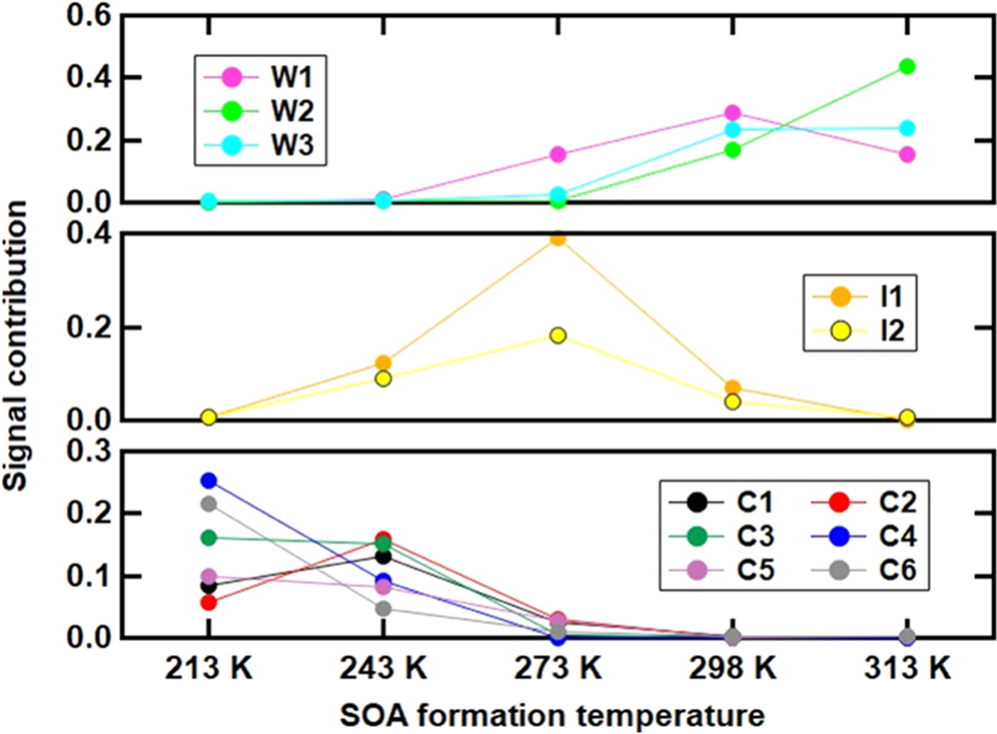
Factor contribution to β-caryophyllene SOA at five formation temperatures. The total detected signals are (3.0 ± 0.9) × 10^4^ counts s^–1^ for each of the five samples.

With varying SOA formation temperatures, PMF factors showed different responses (Figure 3). We grouped the factors according to the behavior of their signal contribution with increasing formation temperatures. “Decreasing factors” (C4, C6) showed lower contributions with increasing formation temperatures. “Increasing factors” (W2, W3) increased their contributions with formation temperatures. “Peak factors” (C1, C2, W1, I1, I2) exhibited first increasing and then decreasing contributions. “Trapezoid factors” (C3, C5) did not change their contribution between the two lowest formation temperatures, but at higher formation temperatures, their contribution decreased.

Contribution of a factor to the measured particle phase composition depends on the contribution of the compounds grouped into this factor that are produced in each experiment. The importance of individual chemical reaction pathways is temperature-dependent; e.g., the degree of autoxidation increases with temperature with a signal fraction of 0.7% of HOM molecules at 213 K increasing to 9.2% at 313 K,^11^ while dimer (C_28–30_H_44–48_O_5–9_) formation was favored at 213–243 K accounting for 53.7 and 32.8% of the signal.^11^ Hence, the particle composition is shifted toward higher oxidized compounds, which have a sufficiently low volatility at higher temperatures. Changes in the contribution of the different factors can also be caused by temperature-dependent partitioning. With higher formation temperatures, the *C*_T_^*^ values of the factors increase, and the gas-to-particle phase partitioning will adjust accordingly. In other words, the compounds grouped into a factor may become too volatile to stay in the particle phase, and the contribution of this factor will decrease with increasing formation temperature.

**Figure 4:**
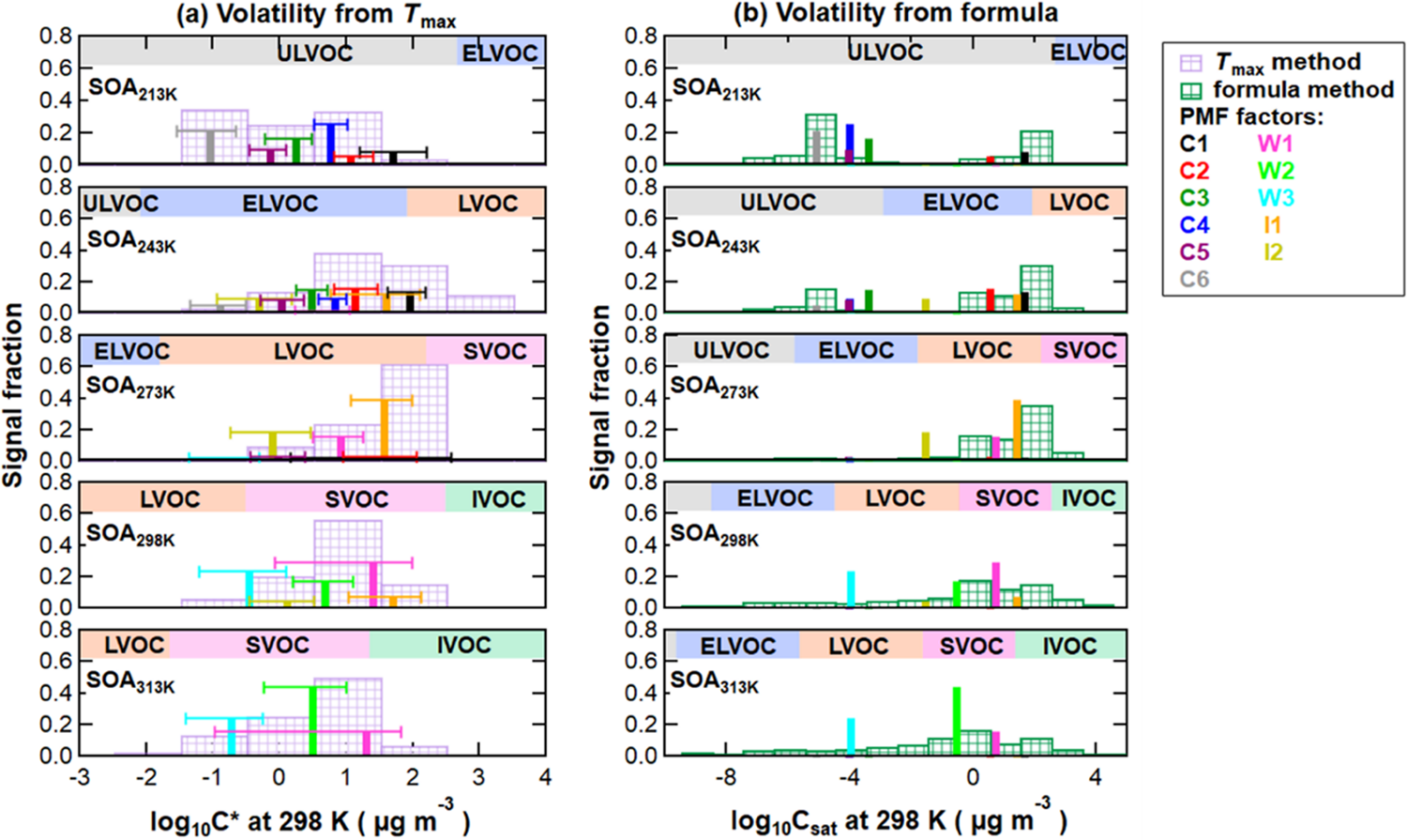
One-dimensional (1D) volatility basis set (1D-VBS) based on the volatility calibration (a), and formula method (b) for SOA formed at temperatures (from top to bottom) of 213, 243, 273, 298, and 313 K. Bars with green and pink grids refer to volatility derived from individual ions (individual thermogram *T*_max_), while solid sticks represent the volatility of factors from PMF analysis (average *T*_max_ for each factor). Note the different *x*-axis ranges in panels (a, b). The colored boxes along the *x*-axis in panels (a, b) indicate the volatility classes:^50,51^ ULVOC, ELVOC, LVOC, SVOC, IVOC, and VOC. These boundaries of the volatility classes are defined for *C*_298K_^*^ and are shifted to the corresponding *C*_T_^*^ values at the formation temperatures using the Clausius–Clapeyron relation.^12^

The decreasing factors (C4, C6) only exist at 213–243 K where all compounds are estimated to be mainly in the ULVOC and ELVOC ranges (Figure 4; volatility prediction is discussed in the next section). The ELVOC and ULVOC categories can be considered to be nonvolatile and hence completely in the particle phase. Thus, the gas-to-particle partitioning did not change between 213 and 243 K and the compounds were only affected by the formation chemistry, indicating that the formation of compounds relevant to the decreasing factors was favored by low temperature. With the same reasoning, trapezoid factors also seem to be mostly governed by the formation chemistry but with a higher optimal temperature (i.e., between 213 and 243 K). Since the peak factors occur over the whole formation temperature range, both mechanisms (temperature-dependent gas-to-particle partitioning and temperature-dependent chemistry) need to be considered. As the partitioning process is expected to have a negligible impact on the factors in the ULVOC range, C1, C2, and I2 are mainly controlled by the formation chemistry with an optimal temperature of 243, 243, and 273 K, respectively. W1 falls into the LVOC (at 273 K) and SVOC ranges (at 298–313 K); thus, the partitioning to the particle phase could be reduced at the highest two formation temperatures. This counteracts the expected increase of the production of compounds grouped into W1 (i.e., HOMs), leading to a peak of the contribution at 298 K. The other two factors (W2, W3) relevant in the warm cases all have lower volatilities (ELVOC to LVOC range). They are increasing factors because the enhanced production with increasing temperature is not affected by changes in partitioning.

Therefore, we emphasize that the impact of temperature on the β-caryophyllene SOA particle formation and volatility is balanced between phase partitioning monotonically and chemical reaction pathways nonmonotonically, leading to different oxidation products existing in the particles with varying optimal temperatures.

Since the FIGAERO-CIMS data provide no direct information about the molecular structure of isomers, we cannot determine the detailed reaction pathways leading to these isomers. However, our study shows that already the β-caryophyllene-derived first-generation oxidation products can produce multiple isomers with volatilities spanning orders of magnitude. Further studies of the molecular structure of such isomers are needed to provide more details of the oxidation processes.

### Volatility Determination and Comparison from Different Methods

For all SOA samples, the volatility distributions derived from the *T*_max_ values of the individual ion thermograms (ion *T*_max_) and the factor thermograms (factor *T*_max_) are displayed in Figure 4a. The *C*_298K_^*^ values are used to facilitate the comparison between samples. Using eq 2, the *C*_298K_^*^ values were converted into the effective volatility class at the formation temperature, which are indicated with colored boxes in Figure 4. Generally, the volatility determined by the PMF factors is distributed at slightly lower values than the volatility derived from the ion *T*_max_ (Figure 4a). The shape of the distribution is similar. This is likely caused by omitting the contribution of isomers and thermal decomposition compounds when using the ion *T*_max_, suggesting the potential overestimation of the volatility of particles containing a range of isomers and thermally labile compounds when using ion *T*_max_.

Based on factor *T*_max_, for SOA_298K_ and SOA_313K_, all factors are in the SVOC range (*C*_298K_^*^ = 10^–0.5^–10^1.7^ μg m^–3^ for SOA_313K_, *C*_298K_^*^ = 10^–0.7^–10^1.3^ for SOA_298K_) (Figure 4a), while for SOA_213K_, the volatility classes shift to the ULVOC range (*C*_213K_^*^ = 10^–1.0^–10^1.7^ μg m^–3^) for both monomer and dimer factors. The significantly lower formation temperatures impact the effective volatility more than the differences in chemical composition. For example, the monomer factors (C1, C2) in SOA_213K_ and SOA_243K_ have higher log_10_* C*_298K_^*^ than the monomeric HOM factor in SOA_298K_ and SOA_313K_ (W2). This confirms that the early-generation compounds (C_14.2–14.9_H_24.0–25.0_ O_4.5–5.6_) are more volatile than HOM species (C_15.0_ H_22.6_ O_6.8_) at the same SOA formation temperatures, e.g., 298 K. However, at the lower formation temperatures, the effective volatilities of C1 and C2 in SOA_213K_ and SOA_243K_ are lower (in the ELVOC and ULVOC ranges) than those of W1 and W2 in SOA_298K_ and SOA_313K_ that are mainly in the SVOC range.

Figure 4b shows the volatility distributions derived with the formula method from the average PMF factor composition and based on the composition of the individual ions. The ion-based values spread a wider range, causing a different shape of the distribution compared with that in Figure 4a. This difference is probably caused by the grouping of ions into the PMF factors and then using the average composition. Thus, values at the upper and lower edges are included in the nearest factor and not as visible as for the individual ion case.

The differences between the formula and the *T*_max_ methods originate from not only the chemical composition (e.g., activity coefficient changes)^48^ in complex chemical mixtures, i.e., SOA particles, but also the existence of thermal decomposition and isomers. This is especially the case for mixtures of compounds with long carbon chains and containing a large fraction of thermally unstable oligomers. The formula method usually predicts too high volatility values because the decomposition products have less carbon and oxygen than the precursors. The observed *T*_max_ value (i.e., maximum of thermal decomposition) is lower than the theoretical *T*_max_ of the precursor, leading to a volatility higher than that of the precursor but lower than the formula method value. Furthermore, the formula method assigns the same volatility to structural isomers. In contrast, the *T*_max_ approach results in different volatilities for structural isomers since the *T*_max_ values vary with chemical structures. Consequently, the discrepancies between the two methods vary for different temperatures because of different amounts of thermal fragments and isomers. For example, for the warmer temperatures (273–313 K), the estimated volatilities are shifted toward lower values when using the formula method. For the colder temperatures (213 and 243 K), additionally, the shape of the distribution changes. Based on the formula method, C4 and C5 have identical volatility, while their *T*_max_ values suggest an order of magnitude difference.

Both the analysis of the key monomers and the PMF analysis indicate that the single ion thermogram can be created by multiple isomers and products of thermal decomposition with a range of volatilities spanning multiple orders of magnitude in C*. Selecting a single *T*_max_ value to represent the volatility of this group of compounds can work well if the group is dominated by one or a few compounds with similar volatilities and the tailing/fronting of the thermogram is not too pronounced. However, it does not account for changes in the ratio between the isomers/decomposition products and may thus overestimate the volatility of the sample.

Overall, our results indicate that the temperature influences not only the partitioning but also the chemical reaction pathways leading to different oxidation products impacting the β-caryophyllene SOA particle formation and volatility. The new volatility characterization based on the PMF analysis of thermogram data suggests that β-caryophyllene oxidation products have a high potential to nucleate aerosol particles and support their growth. Our findings show that the major formation processes for β-caryophyllene SOA vary substantially, depending on the ambient temperatures (e.g., the level in the atmosphere, different seasons, and regions). Therefore, the findings of this work are improving our understanding of the formation of biogenic SOA, e.g., in atmospheric transport models. Further studies on β-caryophyllene SOA formation under other conditions, e.g., daytime chemistry, and its detailed formation mechanisms in gas (e.g., peroxy radical reactions) and particle phases, may unravel the underlying mechanistic changes in more detail.

## Supporting Information Available

Description of the operation of the chamber, the experimental conditions, and the instrument setup for the campaign; discussion of the limitation of offline FIGAERO-CIMS analysis in this work; discussion on wall loss; description of three volatility estimation methods; volatility calibration; O/C, H/C, OS_c_, and *T*_max,sum_ of sum thermograms; van–Krevelen diagram for the bulk particles; comparison of elemental composition and oxidation from the measurements of FIGAERO-CIMS and HR-AMS; gas and particle contribution of C_15_H_24_O_3_; description of PMF analysis of thermograms; diagnostics of PMF solution; VBS based on the formula method for monomer and oligomers; and data related to all figures, individual ions from SOA samples covering the whole temperature range, the volatility calibration, and the information related to PMF factors is openly accessible in the archive KIT (PDF)
