# Supplementary material for: Volatility of Secondary Organic Aerosol from β-Caryophyllene Ozonolysis over a Wide Tropospheric Temperature Range
Source: Environ Sci Technol. 2023 Jun 7;57(24):8965–74. doi: 10.1021/acs.est.3c01151 (PMC10286803; doi:10.1021/acs.est.3c01151)
Supplement: Supplementary file 1 — es3c01151_si_001.pdf [file es3c01151_si_001.pdf]

Supporting Information

Volatility of secondary organic aerosol from β-caryophyllene ozonolysis between 213-313 K

Linyu Gao[1,2], Angela Buchholz[3], Zijun Li[3], Junwei Song[1,2], Magdalena Vallon[1], Feng Jiang[1,2], Ottmar Möhler[1], Thomas Leisner[1], Harald Saathoff[1]**

[1.] Institute of Meteorology and Climate Research, Karlsruhe Institute of Technology, Karlsruhe, 76344, Germany

[2.] Institute of Geography and Geoecology, Working Group for Environmental Mineralogy and Environmental System Analysis, Karlsruhe Institute of Technology, Karlsruhe, 76131, Germany

[3.] Department of Technical Physics, University of Eastern Finland, Kuopio, 70210, Finland

[4.] Institute of Environmental Physics, Heidelberg University, Heidelberg, 69120, Germany

E-mail: linyu.gao@kit.edu, harald.saathoff@kit.edu

Number of pages:19

Number of figures:8

Number of tables:3

Section S1. The operation of the chamber, the experimental conditions, and the instrument setup for the campaign

In the AIDA chamber, air was mixed within 1-2 minutes by a fan which is located about 1 m above the bottom of the chamber [1]. The wall and air temperatures can be controlled with an accuracy of ±0.3 K over a wide temperature range between 313 – 183 K [2].

The data presented in this work were from dark experiments of β-caryophyllene ozonolysis at 213 K, 243 K, 273 K, 298 K, and 313 K, respectively. No hydroxyl radical scavenger or seeds were used. The concentration of β-caryophyllene and ozone, and relative humidity for each experiment are shown in the Table S1. β-caryophyllene (98%, Carl Roth GmbH) was added into a carrier flow of 0.01 m^3 min^{-1} of synthetic air and injected into the AIDA chamber. The concentration in the AIDA chamber was measured by a proton-transfer-reaction time-of-flight mass spectrometer (PTR-TOF-MS 4000, Ionicon Analytic GmbH). Then ozone was generated by a silent discharge generator (Semozon 030.2, Sorbios) with pure oxygen (99.9999%) and injected into the chamber. The O_3 concentration was measured by a gas monitor $O_3$41M (Environment S.A.). In the four experiments between 243-313 K, the initial β-caryophyllene amount was completely consumed, resulting in SOA formation. Subsequently, more ozone was added to accelerate the oxidation of any remaining double bonds in unsaturated β-caryophyllene oxidation products. In the experiment at 213 K, β-caryophyllene was lost to the wall too quickly to be detected and did not lead to significant particle formation after the first ozone addition. Thus, β-caryophyllene was added into the chamber a second time, now in the presence of ozone which then led to the formation of SOA. Note that the excess of ozone was lower for the experiment at 273 K but still about 50 ppb of ozone were available after all β-caryophyllene was consumed. Therefore, we believe the chemistry in this experiment is still comparable to those done at the other temperatures.

We note that the impact of the different RH on our results cannot be completely excluded due to potential condensed phase reactions. However, due to the increasing RH with decreasing T, the potential impact of relative humidity was minimized[3, 4], except for the coldest experiment (213 K).

Table S1. Compilation of experimental conditions, concentration of β-caryophyllene and O_3 (reprinted with permission under the terms of the Creative Commons Attribution 4.0 CC BY License[5]. Copyright 2022 L. Gao).

Exp. No.	SOA particle type	T [K]	RH [%]	β-caryophyllene [μg m-3]	Total O_3 [ppb]	SOA mass+ (SMPS) [$\mu g\ m^{-3}$]

1	SOA_{213K}	213	96	#	320	24.3±6.1
2	SOA_{243K}	243	88	15.8±3.1 (15.6±3.1^t)	317	49.9±12.5
3	SOA_{273K}	273	67	109.5±21.9	73	38.9±9.7
4	SOA_{298K}	298	27	65.0±13.0 (23.2±4.6^t)	325	19.5±4.9
5	SOA_{313K}	313	13	78.6±15.7	290	14.6±3.6

not detectable due to wall loss; t following BCP addition calculated assuming a constant BCP addition rate in each subsequent addition; +SOA mass wall loss corrected.

Section S2. Discussion of the limitation of offline FIGAERO-CIMS analysis in this work

The filter setup and all instruments were installed in the laboratory outside the chamber at room temperature (295±2 K). To minimize the effect of temperature differences between the chamber temperatures (213 K, 243 K, 273 K, 298 K, 313 K) and room temperature on the gas-phase chemical composition, the sampling line to the CIMS was partially insulated and the residence time in the Teflon sampling line was only 2 s. Hence, a significant particle evaporation can be excluded. The residence time in the stainless-steel line for filter sampling was about 1 s. The sampling time of each filter was typically 5-10 minutes plus a few minutes of handling time before the sample was stored in the freezer (-30 °C). The residence time in the stainless-steel sampling line to the AMS was 19 s, very likely allowing the sample air to reach room temperature before entering the instrument. Hence, we cannot fully exclude some particle evaporation and there could be an underestimation of the more volatile fraction of the particles especially in the AMS measurements.

However, high particle viscosity significantly hinders the evaporation of (semi-)volatile compounds from the particle phase. The viscosity of β-caryophyllene SOA particles was found to be in the range between 1.3×10^3 and 2.4×10^7 Pa·s depending on the relative humidity, suggesting β-caryophyllene particles were in a semisolid state[4]. Using a parameterization approach[6], we estimated the particles formed at 213-273 K in this study to be also in a semisolid state. Thus, the diffusivity of β-caryophyllene SOA particles can be assumed to be too low to allow substantial evaporation of particulate compounds during the sampling process.

Section S3. Wall losses (see Gao et al., ACP 2022[5])

Wall losses of particles and semi-volatile trace gases were calculated with the aerosol dynamic model COSIMA[1, 7] and used to correct the SOA yields published already[5]. Particle losses contributed typically 6% or less to the total SOA mass. Due to the large size of the simulation chamber, and the relatively low vapor pressures of the β-caryophyllene oxidation products and the generally high molecular weights of the product spectrum, the diffusion-limited wall loss of gases is of minor importance compared to the condensation onto the organic particle phase. Thus, the losses via the gas phase contribute less than 1 to 6% to the total SOA mass with the highest value at 313 K. At low temperatures (e.g., 213 K and 243 K), besides HOMs, most products are in (or below) ELVOC ranges (see the section "Volatility determination and comparison from different methods" in the manuscript). However, other products with high molecular weights, e.g., $C_{14}H_{24}O_{5-6}$, $C_{15}H_{24-26}O_{3-5}$ and $C_{30}H_{38}O_5$, can still be detected in both gas and particle phases. Thus, we think the effect of wall loss of HOM on the particle-phase chemical composition is limited. Therefore, while the wall losses may differ within this range between the experiments, they cannot explain the distinct changes in composition.

Section S4. Three volatility estimation methods

Formula method. In this work, we predicted the C^*_{298K} of each molecule from its elemental composition applying a parameterization using molecular corridors[8, 9]. The method is the so-called formula method, and can be expressed as[9]:

$$\log_{10}C_0 = (n_C^0 - n_C)b_C - n_O b_O - 2\frac{n_C n_O}{n_C + n_O}b_{CO} \qquad \text{Eq. (S1)}$$

where n_C, n_O are the number of carbon and oxygen atoms in a molecule, and n_C^0, b_C, b_O, b_{CO} are the parameters. For CHO species[9], the values are: n_C^0=22.66, b_C=0.4481, b_O=1.656, and b_{CO}= -0.7790, respectively.

***T*max method.** During the thermal desorption in the FIGAERO, individual molecules with varying volatilities have different thermal responses. The T_{max} correlates to the effective V_p of organic species in a complex SOA mixture[10]. By measuring the correlation between the T_{max} and the vapor pressure of organic species with known V_p, the C^*_{298K} of all the detected molecules can be estimated[10-13]. Here, we calibrated this relationship using eight carboxylic acids which were dissolved in methanol and deposited on the filter with a syringe. The calibration parameters determined are applied to convert the T_{max} value of each detected ion to a corresponding saturation vapor pressure.

PMF factor method. In the PMF factor method, the SOA volatility is not estimated from the individual ions, but from volatility groups (i.e., from the PMF factors). The T_{max} value of each factor thermogram was converted to a C^*_{298K} value using the same calibration parameters as for the T_{max} method. Note that with the PMF factor method, the thermogram signal of a single ion can contribute to multiple factor thermograms (and thus volatility groups) if it, e.g., contains a monomer compound and a thermally decomposed dimer. In contrast, the T_{max} of the thermogram of a molecule or the formula method assigns only one volatility to one detected molecule.

Section S5. Volatility calibration based on the desorption temperature maximum (T_{max}) in the FIGAERO-CIMS thermograms

We calibrated the $T_{max} - V_p$ relationship of the FIGAERO-CIMS instrument using eight carboxylic acids with known saturation vapor pressures (V_p) (Table S2). We choose these compounds to partially cover the vapor pressures of the β-caryophyllene oxidation products. The eight carboxylic acids were mixed in mass mixing ratios, as shown in Table S2. We used three calibrant solutions: a) single acids in methanol, b) mixed acids in methanol with mass ratios simulating β-caryophyllene SOA, and c) mixed acids in methanol with mass ratios simulating monoterpene SOA. The solutions were deposited on PTFE filters using a micro syringe. The reference compounds were thermally desorbed with the same desorption program that was applied to the SOA samples. The T_{max} values obtained for each carboxylic acid are then related to the V_p values available in the literature (Figure S1). Error bars of T_{max} indicate the range of values obtained from measurements of solutions of individual acids and other mixture solutions. The large error bars of the T_{max} suggests high uncertainties among different solutions, potentially stemming from a strong matrix effect. This confirms that different ratios of acids in a solution can induce high uncertainty of T_{max}, and hence bias the determination of volatility. Thus, to get a calibrant T_{max} - V_p correlation that is representative of the β-caryophyllene studied in this work, we used the T_{max} data of acids in the solution (b) and applied the range obtained from T_{max} values from solution (a) and solution (c) as the uncertainty (i.e., as measurement errors) in the linear fitting procedure. This definition of the measurement uncertainties leads to only one-directional error bars for some data points shown in Figure S1.

A linear regression was applied to obtain calibration parameters (r^2=0.8):

$$\log_{10} V_p = -0.48 - 0.04 \times T_{max} \qquad \text{Eq.(S2)}$$

Eq. (S2) was applied to convert T_{max} values of thermograms of individual ions or factor thermograms to the corresponding V_p, and Eq. (1) was used to convert these values to saturation concentrations (C^*_{298K}) using the molecular weight (MW) of the individual ion or the average MW of the PMF factor.

Table S2. Compounds used for the volatility calibration and saturation vapor pressures from literature.

Organic acid	Sum formula	a) Single acid	b) Mixed acids	c) Mixed acids	V_p [Pa]

		mass ratio in mixture (%)	T_{max} (°C)	mass ratio in mixture (%)	T_{max} (°C)	mass ratio in mixture (%)	T_{max} (°C)	from literatures	Reference for vapor pressure
Malonic acid	$C_3H_4O_4$	100	62.0	3	83.6	2.2	78.9	6.2e-4	14
Succinic acid	$C_4H_6O_4$	100	68.6	2	70.2	7.6	65.3	1.3e-3	14, 15
Glutaric acid	$C_5H_8O_4$	100	59.4	2	64.5	14.1	65.3	6.9e-4	14, 15
Adipic acid	$C_6H_{10}O_4$	100	80.9	2	70.2	16.3	78.9	1.8e-4	14, 15
Pinonic acid	$C_{10}H_{16}O_3$	100	82.4	9	51	30.4	42.6	0.01028	16-18
Azelaic acid	$C_9H_{16}O_4$	100	100	3	83.6	16.3	86.6	6.7e-6	15, 19
Tricarballylic acid	$C_6H_8O_6$	100	132	1	155.3	8.7	146.5	3.1e-7*	20
β-caryophyllinic acid	$C_{14}H_{22}O_4$	100	96.2	78	89.5	4.3	98.3	1.3e-8*	21

* values estimated with a group contribution method[20, 21].

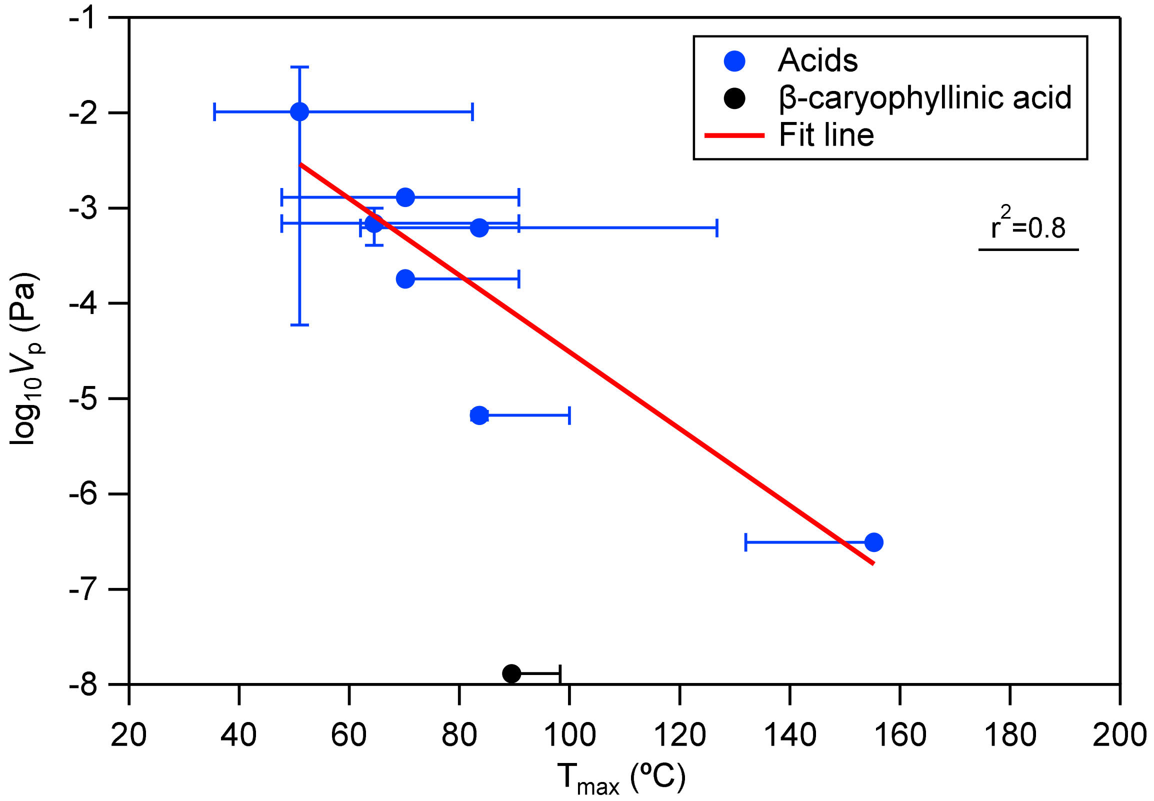

Figure S1. Correlation between temperatures of maximum desorption (T_{max}) and the known saturation vapor pressures (V_P) of seven carboxylic acids. X-axis values for dots refer to the T_{max} of each acid in the mixed acids solution (b) (Table S2). The x-axis error bars values refer to the range of T_{max} of each acid in other mixtures (a) and (c). The β-caryophyllinic acid value is marked with black and was not included in the fit.

Table S3. Compilation of O:C ratios, H:C ratios, OS_C, derived from FIGAERO-CIMS measurements and the T_{max} of sum thermograms.

SOA particle type	O:C	H:C	OS_C	T_{max} of sum thermogram [°C]
SOA_{213K}	0.21	1.64	-1.22	101
SOA_{243K}	0.25	1.67	-1.17	99
SOA_{273K}	0.29	1.70	-1.12	69
SOA_{298K}	0.42	1.71	-0.87	88
SOA_{313K}	0.45	1.66	-0.76	96

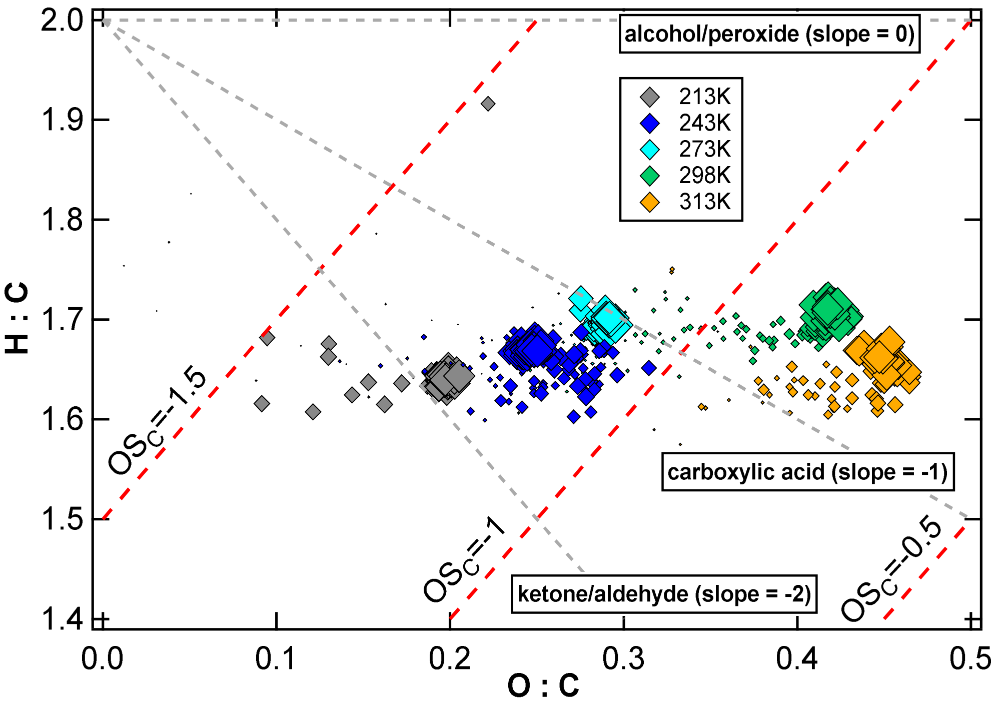

Figure S2. Van-Krevelen diagram for β-caryophyllene-dereived particle bulks during the SOA generation period at all temperatures from HR-AMS measurements. Symbols are coloured by temperature and sized by the time from the first ozone addition. Larger symbols indicates the more aged particles. The carbon oxidation states (OS_C) are shown with red dashed lines. (OS_C= 2 O:C – H:C). Grey dashed lines indicate functionalities that dominate in the SOA particles with data points all along those lines.

Section S6. Comparison of elemental composition and oxidation state from the measurements of FIGAERO-CIMS and HR-AMS

As shown in Figure S3, the O:C ratio and OS_C from FIGAERO-CIMS measurements were slightly higher than those from HR-AMS measurements for all five formation temperatures. Both measurements showed increases in O:C ratio and OS_C of particles with increasing experiment temperatures. The H:C ratios from FIGAERO-CIMS data were generally lower than values obtained from HR-AMS measurements. The discrepancy was interpreted as being linked to the higher sensitivity towards oxygenated molecules with large polarity when using iodide ionisation in CIMS, while HR-AMS detected the bulk particles including low-oxygenated species.

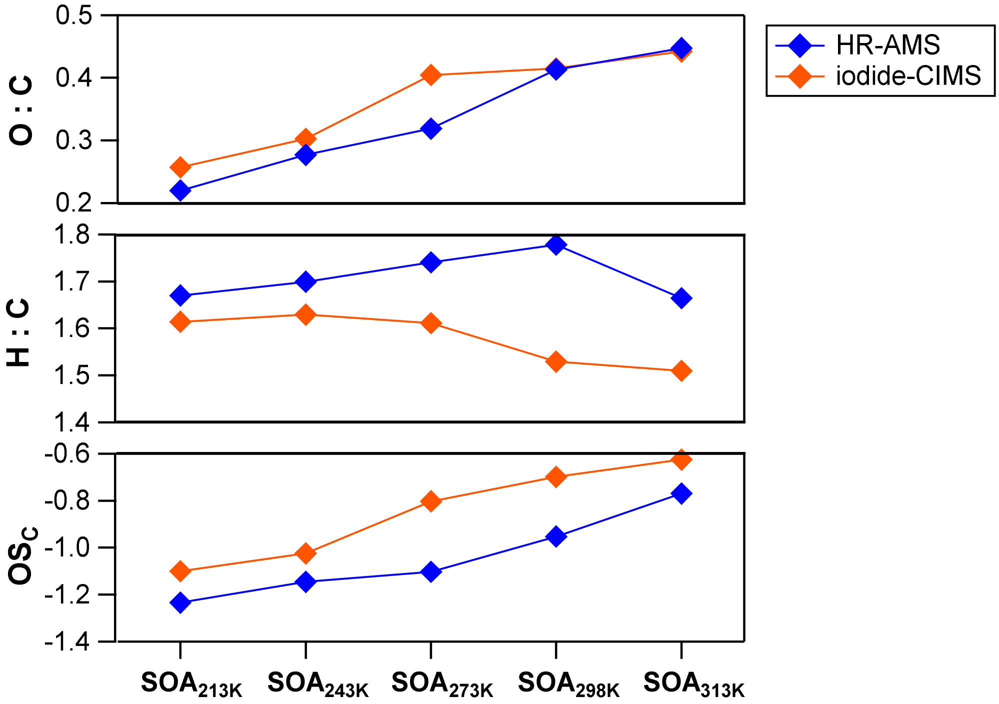

Figure S3. Comparison of O:C ratio, H:C ratio, and oxidation state, OS_C of SOA particles formed at 213 K, 243 K, 273 K, 298 K, and 313 K between the measurements from HR-AMS (blue diamonds) and FIGAERO-CIMS (orange diamonds).

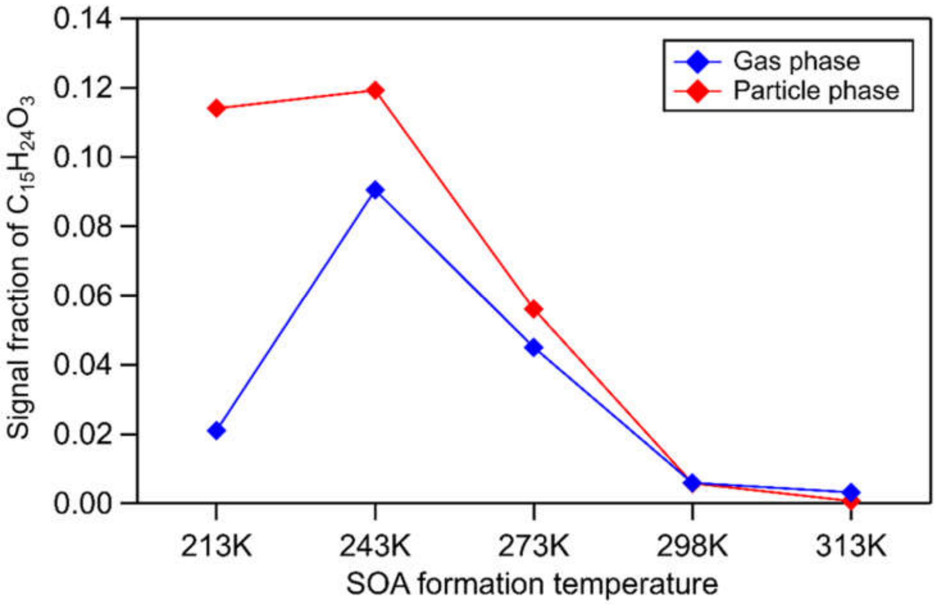

Figure S4. The contribution of $C_{15}H_{24}O_3$ in both gas and particle phases for all SOA formation temperatures. Blue diamonds refer to the gas phase, while red diamonds represent the particle phase.

Section S7. Detailed description of PMF analysis of thermograms

As showed in Figure S5, the two SOA samples in the cold cases are resolved by a similar factor pattern, indicating similar SOA formation processes at 213-243 K. Factor C1 (factor T_{max} of 60 °C) and C2 (factor T_{max} of 85 °C) are dominated by $C_{15}H_{26}O_4$ and $C_{15}H_{26}O_5$, respectively. Those two molecules are the second most abundant monomers after $C_{15}H_{24}O_3$ and $C_{15}H_{24}O_4$ in the cold case [5]. Factor C4 is dominated by the dimer $C_{30}H_{48}O_5$. Another dimer factor for the cold case, C3 has larger contributions of dimers with higher oxidation states (signal-weighted OS_C of -1.0) and correspondingly has lower volatility as indicated by the higher factor T_{max} (105 °C) than C4 (OS_C = -1.2, factor T_{max} = 95 °C). The T_{max} of the dimer factors is higher than the monomer factors as expected due to the longer carbon chain despite their lower mean factor OS_C. In the higher T_{max} region, C5 (T_{max} = 120 °C) shows contributions of both dimeric and apparently monomeric compounds. These apparent monomers may be either thermal decomposition products from larger oligomers, or the monomers with low volatilities. Factor C6 has the highest T_{max} (145 °C) among all cold factors, and comprises of apparently monomeric, dimeric, and trimeric compounds, and is more likely a representation of the mixture of thermal decomposition compounds from oligomers.

Different from the cold case, the two SOA samples in the warm case are resolved by a totally different factor pattern with two monomer factors (W1, W2) and a dimer factor (W3). The chemical composition shows that W2 mainly consists of highly oxygenated organic molecules (HOMs), with a signal-weighted elemental composition of $C_{15.0}$ $H_{22.6}$ $O_{6.8}$, the highest observed OS_C (-0.53) and O:C ratio (0.49) among all factors, and the highest monomer factor T_{max} (100 °C). Thus, W2 is categorized as the least volatile monomer factor. In parallel, as the other monomer factor at the warm case, W1 has the second highest OS_C and O:C ratio (-0.66 and 0.45) among all factors. One molecule containing several isomers in different volatilities, e.g., $C_{14}H_{22}O_{6\text{-}7}$, can contribute to different factors, such as W1 and W2. The PMF analysis identifies those isomers due to a large T_{max} difference of 15 °C between W1 and W2. It signifies that those isomeric monomers belong to different volatility ranges. In addition, the only dimer factor in the warm case, W3, has a high T_{max} of 135 °C and is actually a mix of dimers and again either extremely low-volatile monomers or thermal decomposition products from oligomers.

In the intermediate temperature case (SOA_{273K}), there are only small contributions of the warm and cold-temperature factors while 60% of the signal is explained by the intermediate temperature

factors I1 and I2. These factors differ 55 °C in T_{max} to each other and contain mostly monomers and a small amount of dimers in I2.

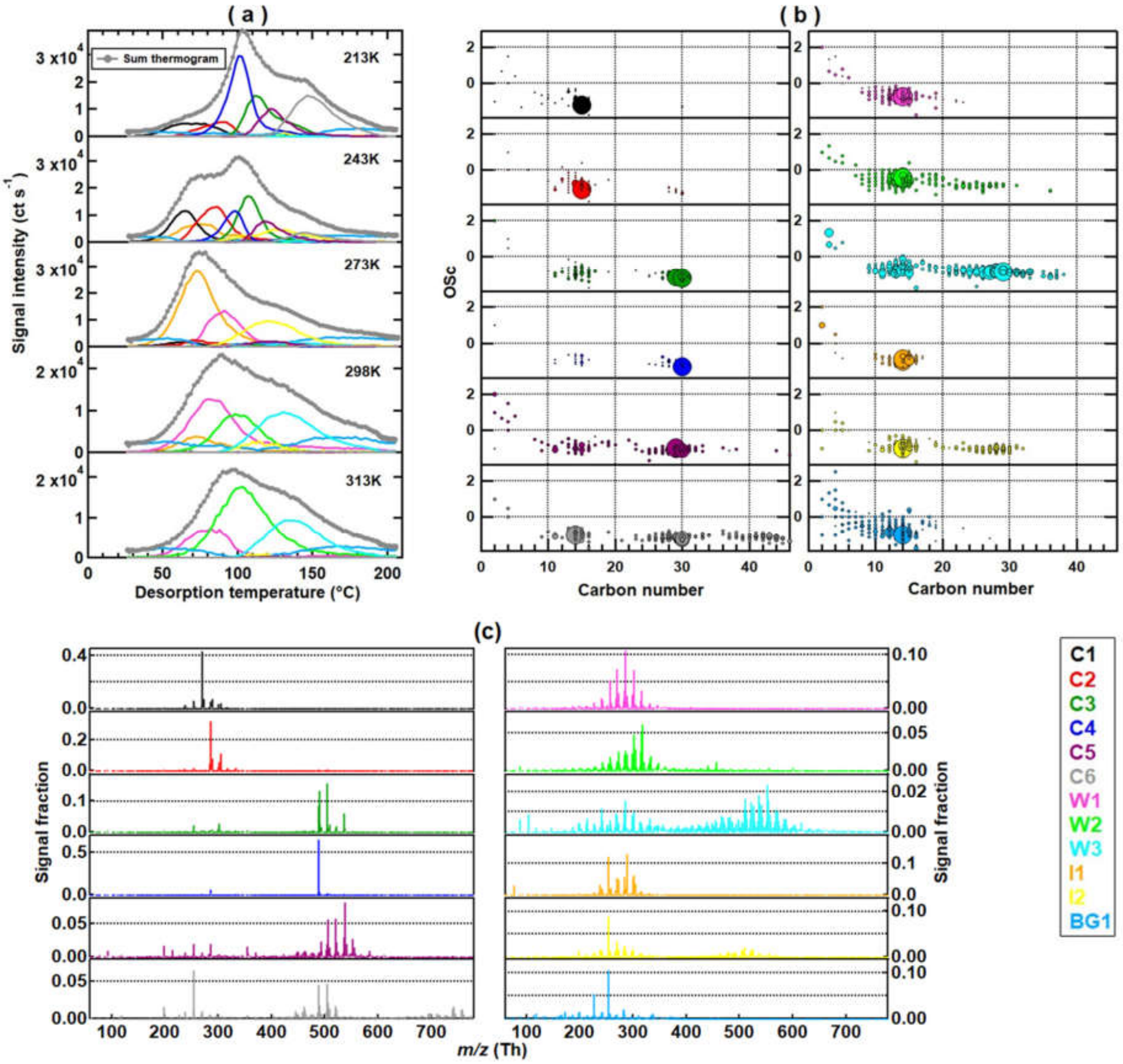

Figure S5. A 12-factor PMF solution for β-caryophyllene SOA particles at five temperatures. (a) sum thermograms (grey lines and points) and the factor thermograms for each sample; (b) the chemical composition of each factor, show as the averaged carbon oxidation state (OS_C) vs the carbon number of each ion (modified Kroll diagram); (c) factor mass spectra. The symbol indicates the normalized signal intensity of each ion. Colors are the same in all panels and indicate the individual factors in (a) , (b) , and (c).

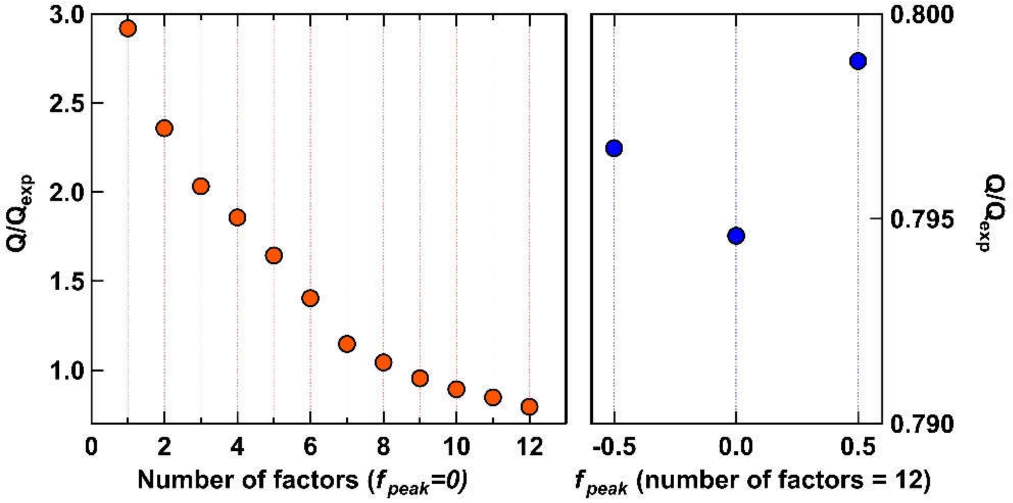

Figure S6. Q/Q_{exp} values for the varying number of factors in PMF solutions (left), and varying f_{peak} in a 12-factor PMF solution (right). Identification of Q, Q_{exp} and f_{peak} have been detailed described previously[22].

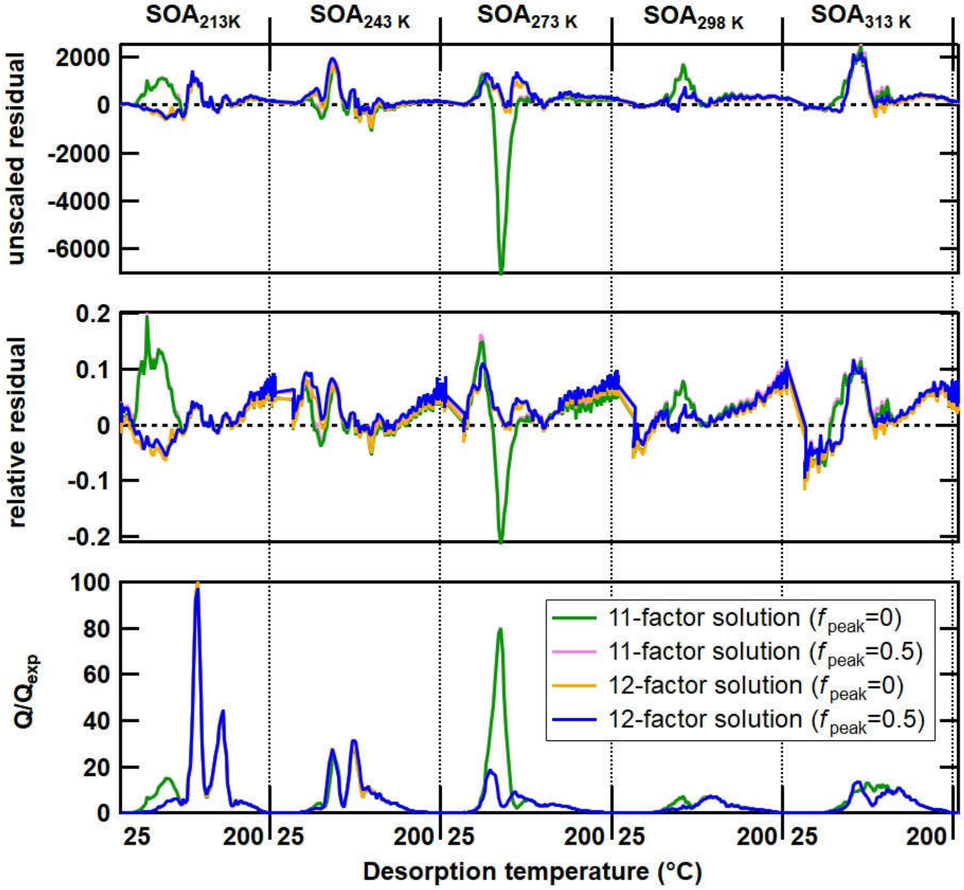

Figure S7. Residual, relative residual, and Q/Q_{exp} change for all samples (SOA formed at 213K, 243K, 273K, 298K, and 313K) when varying the number of factors and f_{peak} values.

Section S8. VBS based on the formula method for monomers, dimers, and trimers.

For SOA_{298K} and SOA_{313K}, molecules span a wide volatility region between the ELVOC and IVOC ranges ($-10 < \log_{10}C^*_{298K} < 6$), with monomers mainly falling into the SVOC and IVOC ranges from the formula method (Figure S8). This range extends to much higher volatilities than the volatility values estimated from thermogram PMF factors or the T_{max} method ($-2.5 < \log_{10}C^*_{298K} < 4$). For SOA_{243K} and SOA_{213K}, the formula approach groups monomeric ions mainly in the LVOC and/or ELVOC classes ($-1 < \log_{10}C^*_{298K} < 4$). For monomers, these predicted volatility values are much higher than those obtained from the PMF factor or the T_{max} method while the values for dimers are in a similar range (ULVOC, $-8 < \log_{10}C^*_{298K} < -3$).

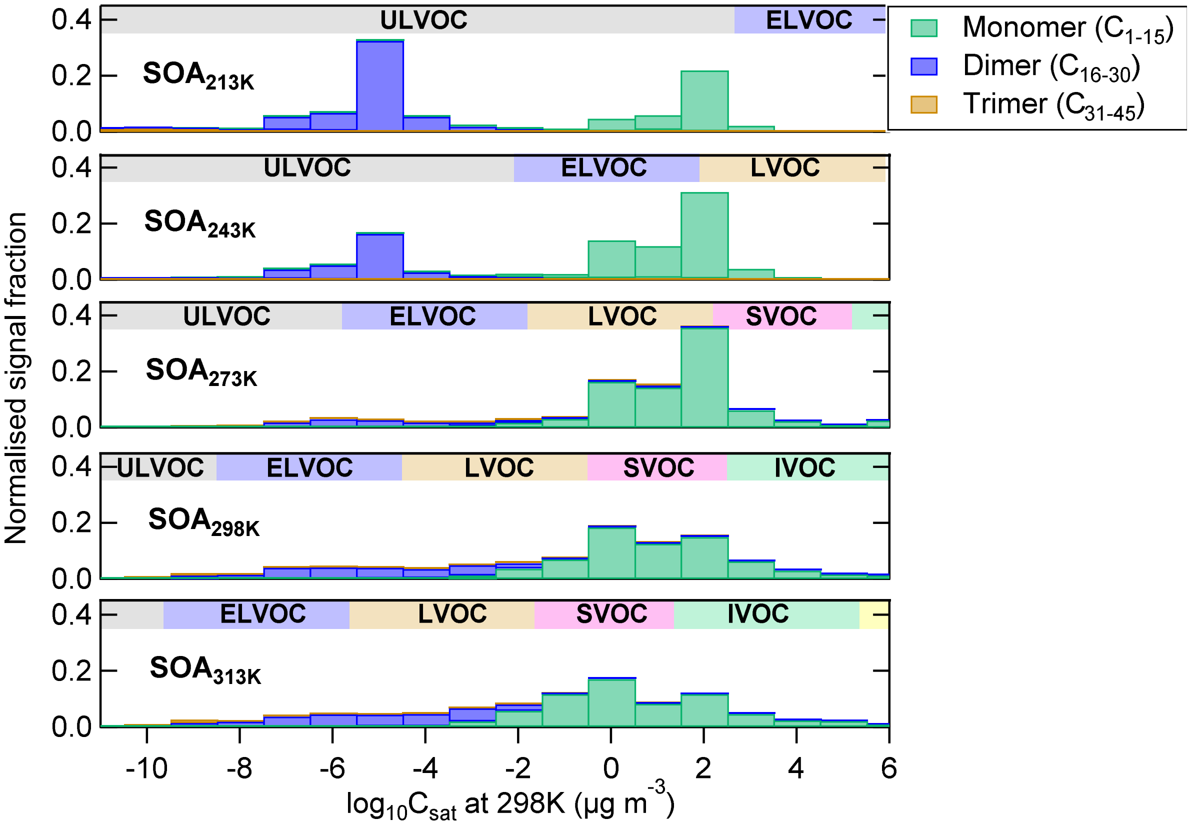

Figure S8. One-dimension volatility basis set (1D-VBS) based on the formula method for monomers, dimers, and trimers in the SOA particles formed at temperatures (from top to bottom) of 213K, 243K, 273K, 298K, and 313K, with bars stack. The colored boxes along the x-axis indicate the volatility classes: ULVOC, ELVOC, LVOC, SVOC, IVOC, and VOC. The boundaries of the volatility classes are defined at 298K and are shifted to the corresponding values at the formation temperatures using the Clausius-Clapeyron relation [23].

References

1. Saathoff, H.; Naumann, K. H.; Möhler, O.; Jonsson, Å. M.; Hallquist, M.; Kiendler-Scharr, A.; Mentel, T. F.; Tillmann, R.; Schurath, U., Temperature dependence of yields of secondary organic aerosols from the ozonolysis of <i>α</i>-pinene and limonene. *Atmos. Chem. Phys.* **2009,** *9*, (5), 1551-1577.

2. Wagner, R.; Bunz, H.; Linke, C.; Möhler, O.; Naumann, K.-H.; Saathoff, H.; Schnaiter, M.; Schurath, U. In *Chamber Simulations of Cloud Chemistry: The AIDA Chamber*, Dordrecht, 2006; Springer Netherlands: Dordrecht, 2006; pp 67-82.

3. Li, Y.; Shiraiwa, M., Timescales of secondary organic aerosols to reach equilibrium at various temperatures and relative humidities. *Atmos. Chem. Phys.* **2019,** *19*, (9), 5959-5971.

4. Maclean, A. M.; Smith, N. R.; Li, Y.; Huang, Y.; Hettiyadura, A. P. S.; Crescenzo, G. V.; Shiraiwa, M.; Laskin, A.; Nizkorodov, S. A.; Bertram, A. K., Humidity-Dependent Viscosity of Secondary Organic Aerosol from Ozonolysis of β-Caryophyllene: Measurements, Predictions, and Implications. *ACS Earth and Space Chemistry* **2021,** *5*, (2), 305-318.

5. Gao, L.; Song, J.; Mohr, C.; Huang, W.; Vallon, M.; Jiang, F.; Leisner, T.; Saathoff, H., Kinetics, SOA yields and chemical composition of secondary organic aerosol from β-caryophyllene ozonolysis with and without nitrogen oxides between 213 and 313 K. *Atmos. Chem. Phys. Discuss.* **2022,** *2022*, 1-44.

6. DeRieux, W. S. W.; Li, Y.; Lin, P.; Laskin, J.; Laskin, A.; Bertram, A. K.; Nizkorodov, S. A.; Shiraiwa, M., Predicting the glass transition temperature and viscosity of secondary organic material using molecular composition. *Atmos. Chem. Phys.* **2018,** *18*, (9), 6331-6351.

7. Naumann, K.-H., COSIMA—a computer program simulating the dynamics of fractal aerosols. *Journal of Aerosol Science* **2003,** *34*, (10), 1371-1397.

8. Donahue, N. M.; Epstein, S. A.; Pandis, S. N.; Robinson, A. L., A two-dimensional volatility basis set: 1. organic-aerosol mixing thermodynamics. *Atmos. Chem. Phys.* **2011,** *11*, (7), 3303-3318.

9. Li, Y.; Pöschl, U.; Shiraiwa, M., Molecular corridors and parameterizations of volatility in the chemical evolution of organic aerosols. *Atmos. Chem. Phys.* **2016,** *16*, (5), 3327-3344.

10. Lopez-Hilfiker, F. D.; Mohr, C.; Ehn, M.; Rubach, F.; Kleist, E.; Wildt, J.; Mentel, T. F.; Lutz, A.; Hallquist, M.; Worsnop, D.; Thornton, J. A., A novel method for online analysis of gas and particle composition: description and evaluation of a Filter Inlet for Gases and AEROsols (FIGAERO). *Atmos. Meas. Tech.* **2014,** *7*, (4), 983-1001.

11. Stark, H.; Yatavelli, R. L. N.; Thompson, S. L.; Kang, H.; Krechmer, J. E.; Kimmel, J. R.; Palm, B. B.; Hu, W.; Hayes, P. L.; Day, D. A.; Campuzano-Jost, P.; Canagaratna, M. R.; Jayne, J. T.; Worsnop, D. R.; Jimenez, J. L., Impact of Thermal Decomposition on Thermal Desorption Instruments: Advantage of Thermogram Analysis for Quantifying Volatility Distributions of Organic Species. *Environmental Science & Technology* **2017,** *51*, (15), 8491-8500.

12. Bannan, T. J.; Le Breton, M.; Priestley, M.; Worrall, S. D.; Bacak, A.; Marsden, N. A.; Mehra, A.; Hammes, J.; Hallquist, M.; Alfarra, M. R.; Krieger, U. K.; Reid, J. P.; Jayne, J.; Robinson, W.; McFiggans, G.; Coe, H.; Percival, C. J.; Topping, D., A method for extracting calibrated volatility information from the FIGAERO-HR-ToF-CIMS and its experimental application. *Atmos. Meas. Tech.* **2019,** *12*, (3), 1429-1439.

13. Ylisirniö, A.; Barreira, L. M. F.; Pullinen, I.; Buchholz, A.; Jayne, J.; Krechmer, J. E.; Worsnop, D. R.; Virtanen, A.; Schobesberger, S., On the calibration of FIGAERO-ToF-CIMS: importance and impact of calibrant delivery for the particle-phase calibration. *Atmos. Meas. Tech.* **2021,** *14*, (1), 355-367.

14. Bilde, M.; Barsanti, K.; Booth, M.; Cappa, C. D.; Donahue, N. M.; Emanuelsson, E. U.; McFiggans, G.; Krieger, U. K.; Marcolli, C.; Topping, D.; Ziemann, P.; Barley, M.; Clegg, S.; Dennis-Smither, B.; Hallquist, M.; Hallquist, Å. M.; Khlystov, A.; Kulmala, M.; Mogensen, D.; Percival, C. J.; Pope, F.; Reid, J. P.; Ribeiro da Silva, M. A. V.; Rosenoern, T.; Salo, K.; Soonsin, V. P.; Yli-Juuti, T.; Prisle, N. L.; Pagels, J.; Rarey, J.; Zardini, A. A.; Riipinen, I., Saturation Vapor Pressures and Transition Enthalpies of Low-Volatility Organic Molecules of Atmospheric Relevance: From Dicarboxylic Acids to Complex Mixtures. *Chemical reviews* **2015,** *115*, (10), 4115-4156.

15. Chattopadhyay, S.; Ziemann, P. J., Vapor Pressures of Substituted and Unsubstituted Monocarboxylic and Dicarboxylic Acids Measured Using an Improved Thermal Desorption Particle Beam Mass Spectrometry Method. *Aerosol Science and Technology* **2005,** *39*, (11), 1085-1100.

16. Salo, K.; Jonsson, A.; Andersson, P.; Hallquist, M., Aerosol Volatility and Enthalpy of Sublimation of Carboxylic Acids. *The journal of physical chemistry. A* **2010,** *114*, 4586-94.

17. Booth, A. M.; Montague, W. J.; Barley, M. H.; Topping, D. O.; McFiggans, G.; Garforth, A.; Percival, C. J., Solid state and sub-cooled liquid vapour pressures of cyclic aliphatic dicarboxylic acids. *Atmos. Chem. Phys.* **2011,** *11*, (2), 655-665.

18. Hartonen, K.; Parshintsev, J.; Vilja, V.-P.; Tiala, H.; Knuuti, S.; Lai, C. K.; Riekkola, M.-L., Gas chromatographic vapor pressure determination of atmospherically relevant oxidation products of β-caryophyllene and α-pinene. *Atmospheric Environment* **2013,** *81*, 330-338.

19. Bilde, M.; Svenningsson, B.; Mønster, J.; Rosenørn, T., Even−Odd Alternation of Evaporation Rates and Vapor Pressures of C3−C9 Dicarboxylic Acid Aerosols. *Environmental Science & Technology* **2003,** *37*, (7), 1371-1378.

20. Nannoolal, Y.; Rarey, J.; Ramjugernath, D., Estimation of pure component properties: Part 3. Estimation of the vapor pressure of non-electrolyte organic compounds via group contributions and group interactions. *Fluid Phase Equilibria* **2008,** *269*, (1), 117-133.

21. Li, Y. J.; Chen, Q.; Guzman, M. I.; Chan, C. K.; Martin, S. T., Second-generation products contribute substantially to the particle-phase organic material produced by β-caryophyllene ozonolysis. *Atmos. Chem. Phys.* **2011,** *11*, (1), 121-132.

22. Buchholz, A.; Ylisirniö, A.; Huang, W.; Mohr, C.; Canagaratna, M.; Worsnop, D. R.; Schobesberger, S.; Virtanen, A., Deconvolution of FIGAERO–CIMS thermal desorption profiles using positive matrix factorisation to identify chemical and physical processes during particle evaporation. *Atmos. Chem. Phys.* **2020,** *20*, (13), 7693-7716.

23. Ye, Q.; Wang, M.; Hofbauer, V.; Stolzenburg, D.; Chen, D.; Schervish, M.; Vogel, A.; Mauldin, R. L.; Baalbaki, R.; Brilke, S.; Dada, L.; Dias, A.; Duplissy, J.; El Haddad, I.; Finkenzeller, H.; Fischer, L.; He, X.; Kim, C.; Kürten, A.; Lamkaddam, H.; Lee, C. P.; Lehtipalo, K.; Leiminger, M.; Manninen, H. E.; Marten, R.; Mentler, B.; Partoll, E.; Petäjä, T.; Rissanen, M.; Schobesberger, S.; Schuchmann, S.; Simon, M.; Tham, Y. J.; Vazquez-Pufleau, M.; Wagner, A. C.; Wang, Y.; Wu, Y.; Xiao, M.; Baltensperger, U.; Curtius, J.; Flagan, R.; Kirkby, J.; Kulmala, M.; Volkamer, R.; Winkler, P. M.; Worsnop, D.; Donahue, N. M., Molecular Composition and Volatility of Nucleated Particles from α-Pinene Oxidation between −50 °C and +25 °C. *Environmental Science & Technology* **2019,** *53*, (21), 12357-12365.
